# Dr. William P. Johnston’s Case of Bright’s Disease—with Remarks

**Published:** 1842-06-25

**Authors:** William P. Johnston


MEDICAL EXAMINER.

NEW SERIES.

No. 26.] PHILADELPHIA, JUNE 25, 1842. [Vol. I.

### *Case of Bright's Disease—with Remarks.*

By William P. Johnston, M. D.

To the Editors of the Medical Examiner.

Gentlemen,—I have the honour to enclose you a case of the disease of Bright which fell under my care some time since. As this disease is but rarely noticed in our medical periodicals, you may, perhaps, deem the case as of sufficient practical importance to merit a place in your valuable journal.

With much respect,
Your obedient servant,
William P. Johnston, M. D.

*Washington, D. C., June 15th,* 1842.

---

### *Chronic Disease of Bright—Nephritis Albuminosa, or Granular Degeneration of the Kidneys.*

(Intermittent Fever for fourteen months — Occasional swelling of face — Dull pain in lumbar region — Subsequently, œdema of ankles — Ascites, and finally general dropsy — Death. At the autopsy, granular degeneration of the kidneys, and enlargement of the spleen — Remarks.)

Lemuel Williams, æt. 20, of good muscular development and temperate habits, removed from the country to this city about two years since and commenced the trade of painter,—during this period has been a member of a fire company, and has on several occasions been exposed to cold and wet. While in the enjoyment of perfect health, Williams was attacked with intermittent fever in August 1840, in consequence of exposure at a fire, as he supposes. This, the first serious disease which he ever recollects to have had, continued for about fourteen months; the paroxysms generally occurred every other day; at times, however, the disease partook of the quotidian type, and during three successive months the quartan.

On no occasion were the chills arrested for more than one week at a time; a physician was never consulted; quinine was not taken.

The period at which the œdema first commenced cannot be accurately determined; his friends say that they recollect his face was sometimes swollen during the spring of 1841. The patient himself first remarked swelling about the ankles in the autumn following, which generally disappeared after walking. Three weeks before Christmas noticed that the abdomen was unnaturally developed; three or four days after observed the swelling of the face, and subsequently the œdema of legs and feet.

Once commenced, the dropsical effusion increased little by little, till finally he could with difficulty see, from the swelling of face and eyelids; the abdomen became immensely distended, and in like manner the legs and feet.

The patient has never had any marked pain in lumbar region; felt a dull pain in this situation whenever he stooped, about a month before swelling commenced. He has never had fever except after chills; appetite has continued natural; thirst moderate; urine has decreased in quantity. He has coughed for about three weeks without expectorating; no soreness of the throat; voice hoarse for a few days only.

The treatment has chiefly consisted of purgative mixtures, prescribed by a quack, which have operated pretty actively, since which swelling in the face has diminished.

*Actual State, February 2d,* 1842.—Decubitus dorsal, inclining to right side, not elevated. Face pale and sallow; moderate emaciation of arms where there is no œdema; intellectual faculties perfect; he is cheerful and contented. Slight swelling of face, chiefly near angle of right maxillary bone, to which side the head is inclined. Abdomen considerably distended; fluctuation perfect. Lower extremities and scrotum increased far beyond their natural size by cellular infiltration; no pain or uneasy sensation. Pulse sixty, natural; skin cool and always dry; appetite very good; thirst considerable; tongue natural; passes about a pint and a half of urine in twenty-four hours; several stools since yesterday. Cough less; dyspnœa moderate. Percussion yields everywhere a natural resonance; no marked dulness over præcordial region. On right side, anteriorly, dulness commences from half an inch to an inch below nipple. In region of spleen dulness commences about two or three inches above lower edge of ribs. Respiration vesicular and expansive, loud over præcordial region. Sounds of heart natural, no "bruit" accompanying them.

As the prepuce offered some impediment to the escape of the urine, in consequence of the œdema, it was punctured in three or four places with a needle.

A purgative, composed of senna, manna, supertart. potass., &c., was administered.

℞. Spts. Æther. Nit. ℥j. Muc. Lem. Lini, Oj.
To drink during day.

*Examination of the Urine.*—The urine is perfectly clear; there is no sediment; when put in a small glass vessel it appears light coloured, not unlike common champaigne. A drop of nitric acid being added, immediately we perceived a cloudy aspect, and after the addition of two or three more drops of the acid, an abundance of yellowish white albuminous flocculi appeared, which speedily sank to the bottom, occupying from the fourth to the third of the space originally occupied by the urine. Heat being applied to a vessel containing a small quantity of urine, a similar albuminous deposit occurred. Acetic acid produced no effect upon the urine. No trace of red globules visible under the microscope.

4th. Passed from one to two pints of urine last night; more than he has heretofore voided in twenty-four hours. Several stools.

Scrotum measures eighteen inches in circumference. Abdomen, on a line with umbilicus, about three feet eight inches. Right thigh, superiorly, about twenty-one inches.

℞. Pulv. Scillæ, gr. ij. Hyd. Chlor. Mit. gr. ss.
Potass. Nit. gr. xij. q. 4 h.

7th. Last night patient was seized with violent pain in penis ; could not sleep.

8 A. M. Countenance anxious ; constant moaning. Prepuce on left side is of a dark brown or blackish colour, presenting a motley appearance ; on same side, penis is red and firm to the feel ; near the root especially, there is a hard red band, which is exquisitely painful to the touch ; the redness embraces a small portion of the scrotum in the vicinity of the pains. Skin rather hot ; pulse frequent.

Bread and milk poultice sprinkled with laudanum to painful part.

In the evening a phlyctæna, a third of an inch in diameter, had formed on left side of prepuce, from which, when punctured, a dark brownish serous liquid escaped.

The inflammation continued to spread until it occupied nearly the whole anterior surface of the scrotum. Superficial gangrene commencing at the prepuce, advanced "pari passu" with the inflammation.

On the eighth, percussion revealed a marked flatness in the posterior and inferior regions of the thorax ; its precise extent, and the character of the respiration could not be ascertained, in consequence of the feebleness of the patient. No flatness over præcordial region. Respiration more frequent and laboured.

On the 9th vomiting came on, and the symptoms became more alarming, and finally the patient died on the 10th, at 1 A. M., up to which time he continued to be perfectly conscious of his situation.

*Autopsy fourteen hours after Death.*

Moderate emaciation of arms and face, which are free from œdema ; no rigidity of extremities ; no vibices ; ascites and anasarca same as during life. Prepuce is partially deprived of its epidermis, and presents a yellowish appearance. Scrotum six inches long by five broad. Superficial gangrene of penis and scrotum. A similar band of gangrene, six inches long by three wide, extends on the right side, from a little above pubis, obliquely upwards and outwards.

*Abdomen.*—When punctured, a quantity of gas escaped from the cavity of the peritoneum. Abdomen contained about two and a half gallons of transparent citron coloured liquid. Peritoneum natural. Intestines not opened.

*Kidneys.*—*Left* is five inches long by two and a half wide, (not weighed.) Externally it is of a pale yellowish colour ; its external coat is easily detached ; the lobules appear rather more distinct than usual ; consistence of kidney good. When divided longitudinally, the cortical portion of the gland is seen to occupy a much larger space than in a healthy kidney ; it is of a yellowish white colour, granulated in appearance, with five short red lines, running from surface towards cones. Cones imperfect ; deficiency supplied by cortical portion.

*Right.*—Four and a half inches long by two and a quarter wide ; thickness one inch and a half, which surpasses that of left. Equally pale externally ; pyramids more perfect ; cortical portion not less abundant, and similar in appearance to that of left kidney.

*Spleen.*—Eight inches long by five wide ; dark brown color internally, and firm.

*Liver.*—Ten and a quarter inches wide ; greatest length seven inches ; right lobe four and a half inches thick. Externally, the liver is of a bluish colour; internally it is dark brown, and contains a quantity of fluid blood.

*Gall-bladder* escaped notice.

*Bladder* contracted.

*Thorax.*—The pleural cavities contain a quantity of yellowish serum; the left about one pint. The pericardium contains a very small quantity of serum, not greater than is usually found. Heart of natural size.

(We were here obliged to discontinue the autopsy, in consequence of the hour appointed for the interment of the body being near at hand.)

*Remarks.*—It is impossible to say with any degree of certainty when the disease of the kidneys actually commenced in this case; that it already existed in the spring of 1841, is highly probable, for it was then first noticed that the face of the patient was occasionally swollen; this was no doubt the commencement of the dropsy, which subsequently existed in so marked a degree.

Dropsy is a symptom which accompanies many diseases, but according as it originates in one region of the body or another, remains local or becomes general; it will be found to aid us much in diagnosis. Thus, for example, in diseases of the heart, œdema first makes its appearance about the ankles and extends upwards. In cyrrhosis of the liver, and in chronic peritonitis, ascites is the form of dropsy which chiefly exists; the extremities being often considerably emaciated, while the abdomen is very much developed. In the disease of Bright, on the contrary, the œdema commences in the face, especially the eyelids, and sooner or later, often with great rapidity, the anasarca becomes general, to which effusion into the several cavities is often superadded—the dropsy thus becoming more general, and the effusion more abundant than in dropsy produced by any other cause. Protracted intermittent fever sometimes terminates in dropsy; this may depend either upon a chronic disease of the liver, chronic engorgement of the spleen, or of an alteration in the whole animal economy; but in no case will the œdema first show itself in the face, nor the effusion become as abundant and as general, as in the case which we have cited, unless the kidneys be also diseased.

Much has been said for and against albuminuria as a diagnostic sign of the disease of Bright. We do not, ourselves, regard it as pathognomonic, though assuredly the most valuable sign of this affection. Albumen, it is true, may *occasionally* be found in the urine of patients who are suffering from various diseases, but it rarely happens, except in the granular disease of the kidney, that the urine yields this principle either abundantly or for any length of time.

In the case of Williams, the anterior history, the absence of any sign of disease of the heart, the dropsy, and the albuminuria, furnished sufficient data to enable us to diagnosticate the affection of the kidneys, and to predict an enlargement of the spleen. The autopsy disclosed to us these two lesions—the granular disease of the kidneys, peculiar to the affection of Bright, and chronic enlargement of the spleen, due to the long continuance of a neglected intermittent fever. The liver, both as respects its size and internal appearance, was such as we are accustomed to regard as healthy; the increased amount of blood in this organ not being in itself sufficient to constitute disease, it would at most but be regarded as a passive congestion. The gall-bladder unfortunately escaped notice, but it probably contained healthy bile.

